# Supplementary material for: Patterns of Intron Gain and Loss in Fungi
Source: PLoS Biol. 2004 Nov 30;2(12):e422. doi: 10.1371/journal.pbio.0020422 (PMC532390; doi:10.1371/journal.pbio.0020422)
Supplement: Table S1 — Also available at http://genes.mit.edu/NielsenEtAl/. (4.3 MB ZIP). [file pbio.0020422.st001.zip › NielsenEtAl/html/364.html]

AN4673.1.NCU09173.1.MG04336.1.FG05679.1


```
 CLUSTAL W (1.82) Multiple Sequence Alignments - Introns Inserted


Sequence 1: AN4673.1	489 aa
Sequence 2: FG05679.1	476 aa
Sequence 3: NCU09173.1	494 aa
Sequence 4: MG04336.1	515 aa
Alignment Length: 532 aa
Number Identitical Residues: 130 aa
Alignment Score (without introns) 7622


MG04336.1 	--------MDFRIPIRIGSSTEALIVPTILVLAAWIIHRRRTVDPG--AGIRFG-SKDNA
NCU09173.1	----MLFTQTFGVSTAHISVAGVIVCLILFTLQRFLTLRLRRTGRERKASIAISKEKDKV
FG05679.1 	MLAIKEFLHSNNVQSTLVLSFCAAACACLGLLVHTLVKRKNLNRYIRVSSPSPEPVKKTA
AN4673.1  	MDIHTGLVFLALVGFCFLFSHSAPQLLARLRGCKTAARTSQTPPRALSPSFSADDKSKTA
          	            :         .                 .       ..      ....

MG04336.1 	PSNK---LVFPPSRRYLVHKLLPNTPTQFEKASFSAIELGPELLAAKALPTSRAPTSDDE
NCU09173.1	KTDLPYLLQFPPSRRHVLATLPG-----FEKVDVQAIT--PEMLKSQALPTVTTPDFDKD
FG05679.1 	--SADYTNVYPPSQRHVLPQISP----EFSSNNVDLSTTR--LLTLDQDYRYANPA----
AN4673.1  	TQSANPATTLPPQLRHVLSTLSLPGDDEYKYKEIPEKEVLRCPLPMTQDYRKCQEE----
          	  .       **. *:::  :      .:.  ..         *                

MG04336.1 	EAGLLTPTGFTLKDVRALGRFPDYPLLSGVPDPEPCGDGFDISKATFRPFRPFRWGYHQT
NCU09173.1	--GFYTPTGFSTQDIKALGRFPDYAALSGVRDPNPVPETWDITKAKFRPYRPFRWGYHQH
FG05679.1 	---AYNFTGFSVDEIKRLGAFTDYAKLSGVPLPTPL-KNFDLDAALPRPYRPFRWAYHQT
AN4673.1  	---RYTPTGFSVAEVKALGDFPDYAALSGVPLPRAYGEKFDVDKALARPYRPFRWAYHQT
          	     . ***:  ::: ** *.**. ****  * .  . :*:  *  **:*****.*** 

MG04336.1 	MA1LMKLEPDYWIELEKNYQASMAARQKLLGQHRDLIMYYEPGSELACRELMEMVVRFLC
NCU09173.1	MA1LMKYDPDWWVELTSSYHTTMATRRALLAQHGSKIFFQNPCASHASRELMEMVLSFLC
FG05679.1 	MS1LKKMDPDFWIEIENTYRPRIIQRQSLYAKHGSDVLQALPGSELACKELMEMVIQFIC
AN4673.1  	MS1LTKMETDWWLELENTYKERIAQRKALFAKHGGGVLAALPGSELACKELMEMALQFYC
          	*: * * :.*:*:*: ..*:  :  *: * .:* . ::   * :. *.:*****.: * *

MG04336.1 	KRYPQYFRLEEDGDE-TILINELLRTRTVVVSSSSSSSSSSSSSSSPPPQHPLEILAATV
NCU09173.1	NRYPQHFSLS--SDQ-TLFHNHLLCTTTHLLQT-----------------DPLQVLFDNV
FG05679.1 	ARYPQAFELHG-----RVFVNHLLGVKQDLDQI-----------------EPLLFLLNHV
AN4673.1  	ARYPQYFSLID----RRIFKNRILGTEQDVRLK-----------------PPLEILMNNV
          	 **** * *        :: *.:* .   :                     ** .*   *

MG04336.1 	PEDFALMLRSDETGLYHLRAALICSSVGWDAGAKRGLSLGDIHGPVVDYRAKMGLGMDR~
NCU09173.1	PEDYAIMMRNETDGLYYLRAAIVCSSVGWYASQHRDAPLKSIHTHVPDYESKMAFSMDR~
FG05679.1 	PEDFALTLRDPATGRYCFRAGVICSSVGWKLSEKIGLGLPEIHAPVPDYKEKMEFSMDR2
AN4673.1  	PEDFGIMMRDDNTGNYFLRAAVICSSMGWNVASKLGLRLDHIHATIPDYKEKMEFSMNR2
          	***:.: :*.   * * :**.::***:**  . : .  *  **  : **. ** :.*:* 

MG04336.1 	YFARLPTDKPIQRCSWTLEDCAPLFSSPGAAAGSEKDGQGGGRGWSRCAVP--EQDLKAE
NCU09173.1	WFSKLSTESPVSRCSWGLEDWEAFHTSP----DPDRSVSGDTMEWTRSAFKGREDEVTVK
FG05679.1 	FFTKMPTDSPIQRGSWGLEVGQPLFIPSE---DPEFRIRESQ-----------NPSLTES
AN4673.1  	FFTKMPADKPIQRGSWGLEVGQPLHTPPG---DPHEKIRESQ-----------DPKLRLE
          	:*:::.::.*:.* ** **   .:. ..    ...     .            : .:  .

MG04336.1 	DVQLRCDWQTLRRLPCSGAVAFNFKAVFTPLSRLRTEPFVPALLATVLREGKPALVVPHK
NCU09173.1	DIKLRCDAQTLRRMPVSGAVVFNFKAIFWDLEELREEKYVPRLLAKVLKEGK-RELMEYK
FG05679.1 	DVHLRVDWQTLRRLPLSGAIVFNFKALFTPISEFRDEPYVPSLVLKVLNEGK-GNILKYK
AN4673.1  	NCYLRTDWQTLRRLPLSAAIVFNFKALFTPITEFRDESCVPALVAKILREGK-KNIMEYK
          	:  ** * *****:* *.*:.*****:*  : .:* *  ** *: .:*.***   :: :*

MG04336.1 	AVGHVRELVLPVLDGWAEEQVEEGVVPRGWEAGTLDEHPFYPGWEERWRAEQGF
NCU09173.1	CEEHVRQVAVRACEQWAEEQVEKGWVSREWEVGTLEESPFFPGWEGKWRGEQGF
FG05679.1 	GTWHVEHVAKPALRRYEREQIDKGLIPGDWQTTTLDENPFFPGWEKKWNMS---
AN4673.1  	GTWHVEHLVLPNLDIWAREQEENGLAPKNWEVATLDESPWFKGWEEKWHRQQGF
          	   **..:.      : .** ::*  .  *:. **:* *:: *** :*. ...
```
